# A community-based strategy for the prevention of gender-based violence with people who live in vulnerable conditions in Seville, Spain

**DOI:** 10.3389/fsoc.2025.1643070

**Published:** 2025-10-06

**Authors:** Mahshid Deldar-Abad-Paskeh, Fátima Morales, Isabel María Pedraza-Romero, Nina Salimi-Leisner, Susana Sánchez-Fidalgo, Carmen García-Gil, Antonia Ávalos-Torres

**Affiliations:** ^1^Department of Preventive Medicine and Public Health, University of Seville, Seville, Spain; ^2^Organization Mujeres Supervivientes, Pumarejo Home, Seville, Spain

**Keywords:** gender-based violence, migrant women, people who live in vulnerable conditions, Spain, community-based strategy, prevention, health intervention, public health

## Abstract

**Introduction:**

Gender-based violence is a global problem, present in various spheres: economic, political, social, health-related, and cultural. According to official data, in 2021, there were 87,307 calls to the 016 helpline, 120,813 reports of gender-based violence, and 44 women were murdered due to gender-based violence in Spain. The prevalence and differences in the probability of experiencing gender-based violence in Spain show an unequal distribution between Spanish and migrant women, with the latter being in a more vulnerable situation. This study evaluates a community intervention strategy for the prevention of gender-based violence in people who live in vulnerable conditions.

**Methods:**

A mixed-method study was conducted, using an adapted questionnaire as well as personal open-ended interviews with each participant. The participants were adults attending the *Casa Pumarejo* soup kitchen in Seville, including both Spanish and migrant women aged between 20 and 50 years. Additionally, their prior training in gender equality and their knowledge of violence detection were assessed. In personal interviews, an in-depth exploration of each woman’s lived experience and their understanding of how to act or prevent gender-based violence was carried out.

**Results:**

Before the intervention, the participants did not perceive gender-based violence, as they had not received training on the subject, except for those whose studies were related to gender equality. During the intervention, it was discovered that the association *Mujeres Supervivientes* had developed a successful methodology for assisting people who live in vulnerable conditions and had experienced gender-based violence. Notably, most participants did not know where to seek help when experiencing violence, nor were they aware of the 016 helpline.

**Discussion:**

Although this is a small-scale local initiative, we can conclude that, thanks to this intervention, the women have improved their ability to detect and prevent gender-based violence.

## 1 Introduction

**Figure 1:**
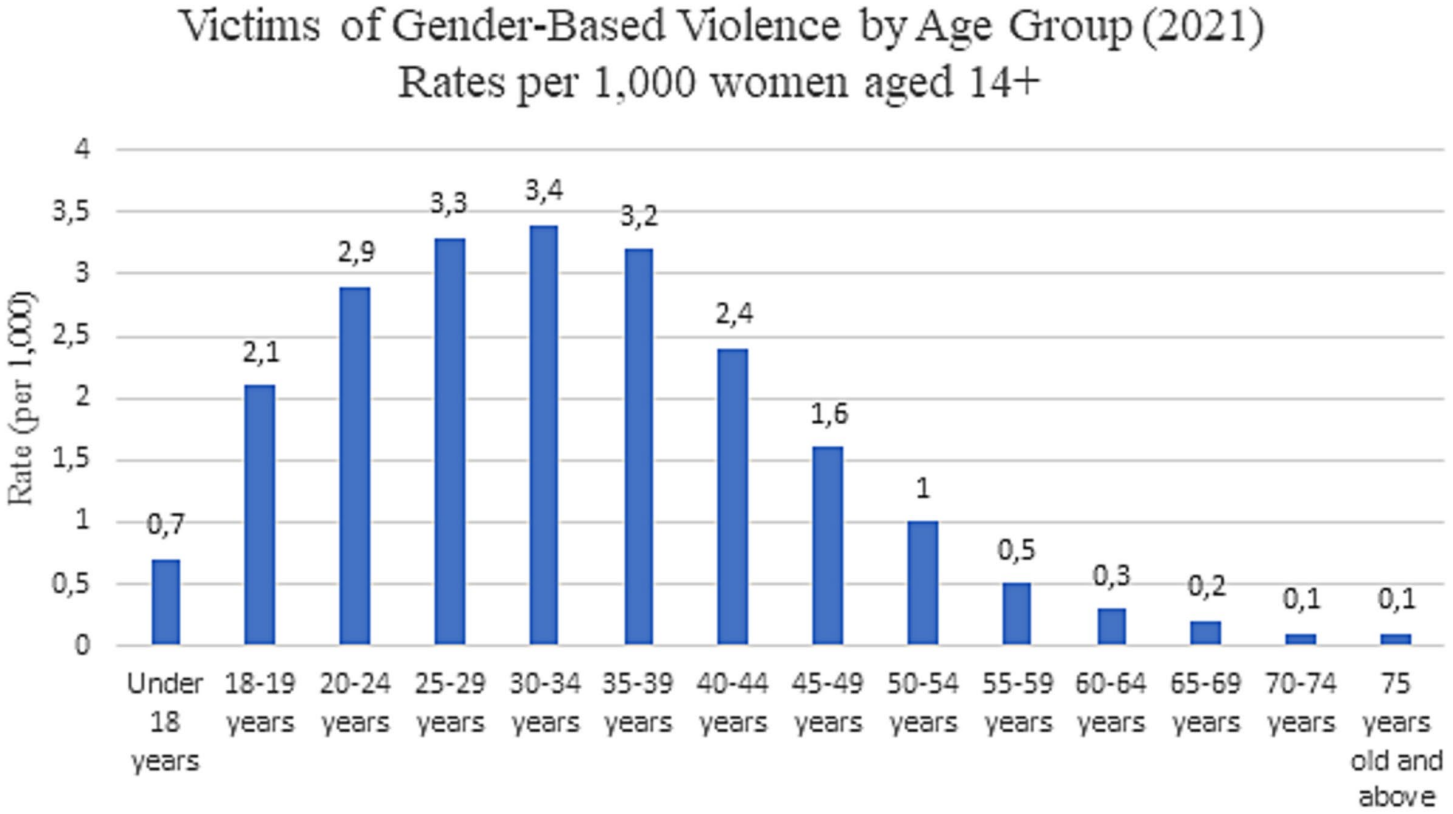
Victims of gender-based violence by age group in 2021 (Instituto Nacional de Estadística, 2021).

**Figure 2:**
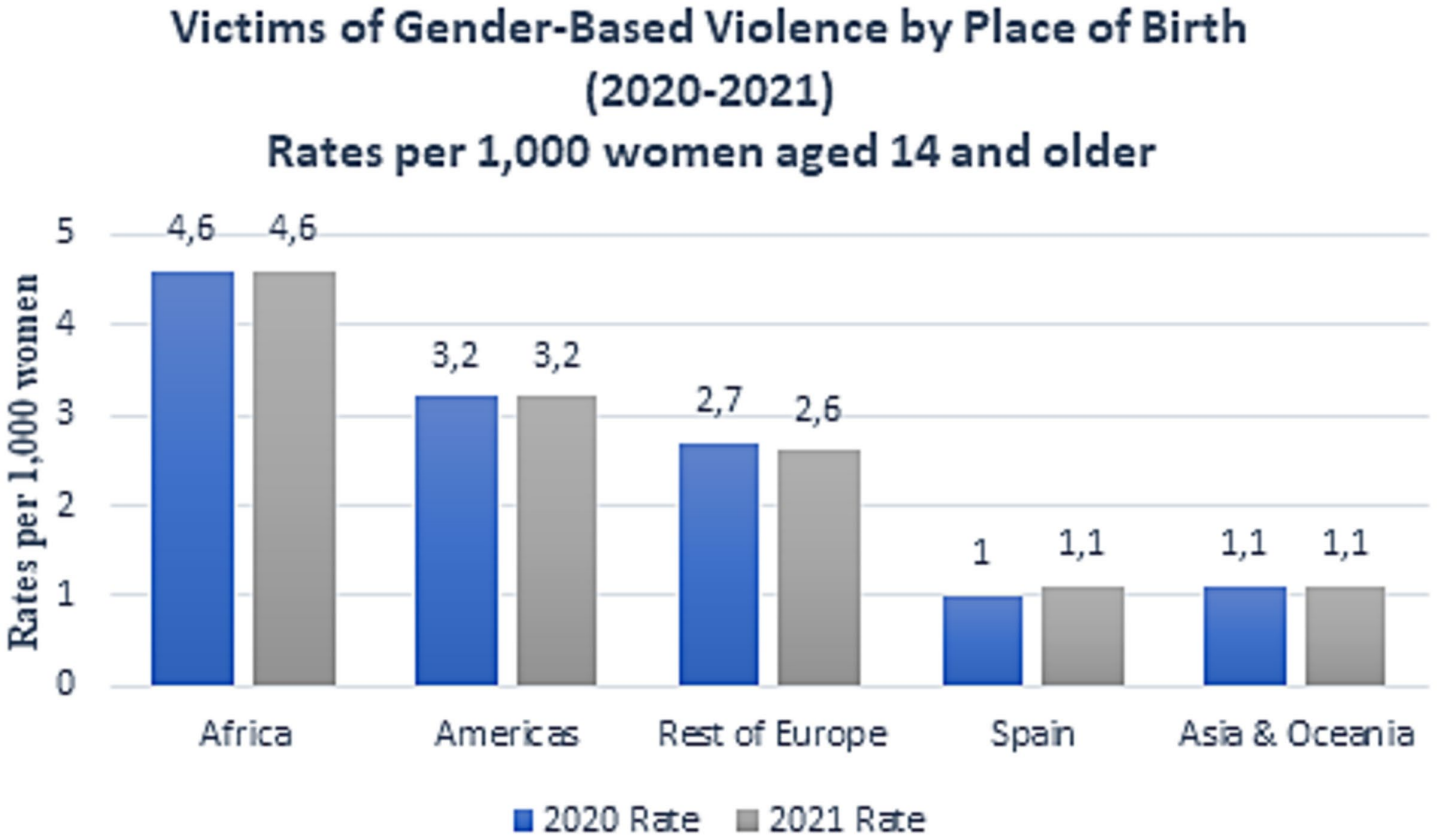
Victims of gender-based violence by country of birth in Spain, 2021 (Instituto Nacional de Estadística, 2021).

In Spain, according to a press release from the National Statistics Institute (Instituto Nacional de Estadística, 2021), the number of female victims of gender-based violence increased by 3.2% in 2021, reaching a total of 30.141 women. Spanish institutions define gender-based violence as any act of physical or psychological violence perpetrated against a woman by a man who is or has been her spouse or is or has been in a similar affective relationship with her, even without cohabitation (Instituto Nacional de Estadística, 2021). Figure 1 shows that the average age of victims was 36.9 years, though the sharpest increase in victim numbers in 2021 was observed among women under 18 (+28.6%). The victimization rate stood at 1.4 per 1,000 women aged ≥14. However, the highest rates were recorded in the 30–34 age group (3.4 victims per 1,000 women) and the 25–29 age group (3.3 victims per 1,000 women) (Instituto Nacional de Estadística, 2021). Migrant women are more likely to experience gender-based violence than Spanish-born women (3.1 per 1,000 migrant women compared to 1.1 per 1,000 native women), with the highest prevalence among women from Africa and the Americas (Figure 2). The autonomous community with the highest number of reported gender-based violence cases in 2021 was Andalusia (6,720 victims) (Instituto Nacional de Estadística, 2021).

The heteropatriarchal system generates sexist behaviors-assigning specific roles to individuals based solely on their gender as women or men. Patriarchy rest on a gender binary system in which only two genders exist, men dominating women (Smith, 2016). Sexism comprises two types: hostile/traditionalsexism and benevolent/modern sexism. Hostile Sexism constitutes prejudiced attitudes or discriminatory behaviors rooted in presumed female inferiority or difference. According to Expósito et al. (1998), this manifests through three dimensions: (i) Dominative paternalism: Belief that women are weaker and inferior to men, (ii) Competitive gender differentiation: Perception that women lack traits required for social governance, relegating them to domestic spheres, and (iii) Heterosexual hostility: View of women as dangerous manipulators through “sexual power.” With the emergence of gender studies in Social Psychology, researchers analyzed the psychosocial consequences of traditional sexism. Findings revealed that defining sexism solely as negative attitudes toward women fails to capture its prevalence in Western societies. This insight led to the conceptualization of ambivalent sexism (Expósito et al., 1998), comprising both hostile and benevolent forms. Benevolent Sexism refers to interrelated attitudes that stereotype women within limited roles yet carry positive affective tones, often eliciting prosocial or intimacy-seeking behaviors (Expósito et al., 1998). Glick and Fiske (1996) argue this form may be more harmful than hostile sexism. Also, Hammond et al. (2017) highlighted that benevolent sexism is particularly tenacious. Benevolent sexism operates through three components (Expósito et al., 1998): (i) Protective paternalism: Men “safeguard” women as fathers protect children, (ii) Complementary gender differentiation: Framing female traits as “positive” yet merely supplementary to male traits, and (iii) Heterosexual intimacy: Paradoxical dependence of dominant-group males on subordinate-group females. However, all forms of sexism have negative effects on how women are perceived and treated by others as well as on women themselves (Barreto and Doyle, 2023). The Glick and Fiske’s Ambivalent Sexism Inventory (ASI) was adapted into Spanish by Expósito et al. (1998).

From an early age, social discourses promote the idealization of romantic love as the exclusive path to female fulfillment. This is what romantic love reveals, causing women to remain in the background, dedicating themselves to caring for and loving others while tolerating any type of violence. For Sternberg (1986), romantic love derives from the combination of intimacy and passion, lacking of the essential decision/commitment component to complete the triangular theory of love. Idealization of love and romantic myths are very common because of culture and society. However, love myths can be a condition factor to develop dysfunctional relationships and a risk factor for gender violence (Martín-Salvador et al., 2021; Jiménez-Picón et al., 2023). Gender differences in love-role definitions contribute to male dominance in intimate relationships (Heiss, 1991). In fact, among girls aged 16 to 24 who have had a romantic relationship, 2.5% experienced physical violence, while only 0.8% of those over 25 experienced it. Additionally, girls who experienced it show more difficulties in their studies, integration with peers, and have low self-esteem, causing them mental, physical, and social health problems (Martín-Salvador et al., 2021). These data reflect not only a structural issue but also the need to understand the psychosocial factors that perpetuate gender-based violence. The first scale developed was the Domestic Violence Myth Acceptance Scale (Peters, 2008), but it had some psychometric limitations, so a new scale called “Acceptance of Myths about Intimate Partner Violence against Women” (AMIVAW) was developed. One version was created in Spanish and another in parallel in English (Megías et al., 2017) to observe the similarity of myths integrated by Spaniards and Americans regarding gender-based violence. It was studied whether each item met the following criteria: (i) representativeness, (ii) comprehension, (iii) ambiguity and (iv) clarity. This scale was positively correlated with scales measuring similar issues, including attitudes toward domestic violence and sexual assaults, considering Spanish and American samples. As predicted, high scores on the AMIVAW scale (Megías et al., 2017) were inversely related to feminist ideology and independent of sexual attractiveness, and were directly related to hostile sexism and, to a lesser extent, benevolent sexism. Currently, this scale is a useful tool for detecting common myths about violence committed by men against women in intimate relationships (Megías et al., 2017). It has also been adapted to other languages such as Portuguese (Giger et al., 2017) and French (Courtois et al., 2021).

One of the tools that could be used to prevent gender-based violence is detecting it from the healthcare sector. For this, healthcare personnel must be trained on how to treat a patient suspected of suffering gender-based violence. Currently, in developed countries like Australia, 80.9% of nurses do not routinely include gender-based violence surveys in patient assessments. Only 50.2% of nurses ask directly when they think there is a chance the patient is suffering from it (Aljomaie et al., 2022). The reasons for this could be: lack of training for healthcare professionals, lack of privacy in emergency rooms, lack of trust from the victim, fear of consequences (Aziz and El-Gazzar, 2019). All these factors make healthcare personnel reluctant to address this issue with patients without knowing how to manage it properly (Aljomaie et al., 2022; Aziz and El-Gazzar, 2019). Two strategies implemented to prevent gender-based violence were conducted in developed countries through their healthcare systems. In both, healthcare professionals were trained to identify and address gender-based violence in their patients, though in England it targeted all women in a specific area (Feder et al., 2011), while in Australia it targeted only refugee and migrant women (Taft et al., 2021). However, another study from Tanzania evaluated the impact of gender-based violence on women in rural areas by including them in a microfinance system (Harvey et al., 2018) with the goal of bringing groups of women together to feel confident and empowered to share their experiences about gender-based violence. All interventions were useful as they increased the detection of gender-based violence. In Tanzania, women disclosed their situations more openly and concealed the violence they suffered less (Harvey et al., 2018). In developed countries, thanks to the training received by healthcare professionals, the rate of identification and detection of gender-based violence among their patients has increased (Feder et al., 2011; Taft et al., 2021).

Therefore, gender-based violence is a current public health issue that affects many women worldwide. Some of the underlying causes of this violence stem from society’s perception of romantic love and sexist attitudes. To prevent it, effective strategies must be implemented through healthcare networks or other tools, such as the rural empowerment of women. *Mujeres Supervivientes* is an organization of professional women with the mission of building a society free from gender-based violence, fostering equality, democracy, and the coexistence of all people. It emerged from the need for survival and support for women experiencing all forms of violence and discrimination, rooted in intersectional feminism. Their work involves holistic care tailored to the needs of women, delivered through services such as legal counsel, emotional support, case management, and personalized accompaniment (Mujeres Supervivientes, 2025). So, the main objective of this study is to evaluate a community intervention strategy for the prevention of gender-based violence in people who live in vulnerable conditions, developed by *Mujeres Supervivientes*, an association located at *Casa Pumarejo*, a community center in Seville, Spain. To achieve this, we planned to: (i) Characterize the population participating in the intervention, (ii) understand the prevention and treatment methodology for gender-based violence situations used by the association, (iii) assess the prior knowledge and experiences regarding gender-based violence of the people attending the center and (iv) analyze the knowledge acquired after the community intervention to prevent gender-based violence.

## 2 Methods

### 2.1 Study type and participants

It is a cross-sectional descriptive observational study that includes a mixed analysis. The study involved 20 women in the quantitative analysis and 8 women in the qualitative analysis. The participants were women attending the *Casa Pumarejo* social dining hall: (i) volunteers and staff of the *Mujeres Supervivientes* association, (ii) students doing their university or vocational training internships there and (iii) women interested in attending the gender-based violence prevention workshops offered. The inclusion criteria were: adult women (with 18 years of age or older), providing informed consent and willingly agree to participate in the study, with ability to understand study procedures, consent forms, and intervention materials, and to communicate effectively in Spanish, residing in Seville, Spain. Key social determinants of vulnerability, such as housing insecurity, caregiving responsibilities, migration status, and economic precarity, were taken into consideration as essential dimensions of the analysis for understanding the complexity of gender-based violence.

### 2.2 Methodological instruments

#### 2.2.1 Quantitative study

A survey was conducted at the beginning and at the end of the intervention. The script for the survey was adapted from a questionnaire carried out by the Unit for Equality at the University of Seville, which used various scales. The ones selected and adapted to our study’s objective are detailed below:

- Scale on the Acceptance of Myths about Intimate Partner Violence Against Women (AMIVAW) (Megías et al., 2017). Considering sociocultural factors, this scale measures the relationship between blaming the victim and exonerating the aggressor. It consists of 15 Likert-type statements, allowing one to gauge the level of disagreement or agreement with a statement. It uses a 7-option scale (1 = strongly disagree; 7 = strongly agree). To assess the reliability of the test, Cronbach’s alpha was measured, which evaluates the correlations between the variables forming the scale to determine its consistency. This coefficient ranges from 0 to 1; the closer it is to 1, the more consistent the items are with each other, and thus the higher the reliability of the scale. In this case, the Cronbach’s alpha is 0.93, indicating high reliability.
- Another scale deals with the Justification of Sexism and Violence (Díaz-Aguado Jalón, 2005), measuring the relationship between emotional intelligence and the understanding of emotions concerning sexism, and also studying other concepts like the concept of partner and gender violence. This questionnaire consists of 12 Likert-type items with 4 response options (1 = strongly disagree; 4 = strongly agree). The scale includes two factors that provide information about the degree of justification of gender-based violence and the patriarchal family (factor 1, based on 7 items) and the degree of justification of sexism and violence as a reaction (factor 2, with 5 items). The scale shows good reliability (Cronbach’s alpha = 0.83).
- A final scale is based on the Ambivalent Sexism Inventory (ISA) (Expósito et al., 1998) to measure the degree of hostile sexism (related to negative attitudes toward women) and benevolent sexism (related to positive, yet discriminatory attitudes toward women, formed by three different aspects: protective paternalism, complementary gender differentiation, and heterosexual intimacy). It consists of 22 items, 11 for each type of sexism, with 6 response options (0 = strongly disagree; 5 = strongly agree). It is a widely used instrument due to its high reliability (Cronbach’s alpha of 0.92).

Additionally, several sections were included to gather sociodemographic data such as age, sex, postal code, professional situation, marital status, etc. Due to the intentionally small sample size of 20 participants, traditional inferential statistical tests (e.g., t-tests, ANOVA) and the calculation of confidence intervals were deemed inappropriate. The analysis primarily focuses on descriptive statistics, specifically presenting frequencies and percentages.

#### 2.2.2 Qualitative study

The qualitative research conducted is based on a narrative phenomenological methodology, which delves into how people perceive their environment and their own professional reality concerning gender violence in general and the care for children exposed to gender violence in particular. Phenomenology (Cerbone, 2012) can be particularly useful for interpreting the facts and processes studied, capturing the meaning of phenomena and the intent behind social activities. This is why it is important for this study, as the voices of the participants are a fundamental source of analysis. Throughout the qualitative research process, reflexivity is employed to ensure the rigor of the study. This technique is commonly used in feminist articles, publications, and other works, as it moves away from the ideas of objectivity and detachment so valued in the positivist paradigm; it expresses the researcher’s awareness and connection to the research situation. Thus, qualitative analysis was carried out because it provides more complete and in-depth information (Morales, 2021), helping to clarify doubts, obtain more pragmatic answers, and even discover new research avenues during the process. Among the most commonly used techniques in qualitative research, we chose in-depth interviews, as they allow for direct and spontaneous face-to-face dialog with the participants, with a high level of communicative intensity. This interview was designed specifically for the study, reviewed and validated by professionals in research methodology and gender studies from the Unit for Equality at the University of Seville.

The script consists of theoretical categories and open-ended questions to address the following topics: (i) knowledge, beliefs, and stereotypes the participants have about gender violence: a series of questions are asked about definitions and basic concepts related to gender violence, (ii) models for the transmission of myths and prejudices on the subject: questions are posed about factors that may or may not influence gender violence and characteristics of the population that exercises and suffers from it, (iii) feelings and emotions about gender violence: information is gathered on how participants feel and what gender violence provokes in them, (iv) training received in gender violence: refers to any training proposals that the participants suggest for preventing gender violence and (v) experiences of gender violence: in this section, participants share any personal experiences they may have had with gender violence, if they are willing. For the interview conducted after the final workshop, an additional topic is included: (vi) opinion on the workshops delivered: to evaluate the usefulness of the workshops, questions were asked about what the participants thought of them and any improvements they would suggest.

The interviewer used a recorder, with the prior consent of the participants. They were kindly asked at the beginning of the interview in a friendly and approachable manner. This also helped “break the ice” and gain the participants’ trust. Once consent was given, the device was placed in a non-visible area to ensure it did not disturb the participants during the interview, thus avoiding unnecessary biases in the research. Each participant chosen for the interview was recorded with their consent, being informed that their data would be used for research purposes and might be published in academic works, while ensuring confidentiality and anonymity. The interviews were conducted at *Casa Pumarejo* and lasted approximately 15 min each.

The processing of all information obtained from the interviews, along with discourse analysis, has been structured into three stages: (i) transcription of interviews, (ii) categorization of content, and (iii) discourse analysis and reflection. To minimize biases inherent in qualitative research, a triangulation approach was adopted, considering sources, methods used, and both verbal and non-verbal aspects of the informant’s discourse. The qualitative data obtained from the interviews underwent a rigorous coding process, systematically deconstructing the raw textual data into meaningful units. This iterative process began with open coding, where initial categories and concepts emerged directly from the data without preconceived notions. Subsequently, axial coding was employed to identify relationships between these categories, leading to the development of broader themes. Finally, selective coding refined these themes into a cohesive and comprehensive thematic structure, representing the core insights derived from the data. The analytical framework underpinning this study was primarily inductive, allowing themes and patterns to emerge organically from the data rather than imposing pre-existing theoretical constructs. This approach ensured that the findings were deeply rooted in the empirical data, enhancing their trustworthiness and authenticity.

#### 2.2.3 Stage planning

The project was divided into four stages:

##### 2.2.3.1 Ethnography of the location

The intervention site is located in Spain, specifically in Andalusia, in the city of Seville, in the *San Gil* neighborhood of the historic district (*Casco Antiguo*). More precisely, it is situated in the *Plaza de Pumarejo*, named after the prominent house located there “the *Casa Grande del Pumarejo*.” This is an old palace-like house originally intended for conversion into a high-end hotel. However, local residents mobilized to protect the heritage value of the house, successfully securing its inclusion in the General Catalog of Andalusia’s Historical Heritage in 2003 as a monument, declaring it a Site of Cultural Interest (*Bien de Interés Cultural*), thereby preventing its privatization. Currently, the property belongs to the City Council, which has an agreement with the *Casa de Pumarejo* Association until 2026, allowing them and other neighborhood associations to conduct activities there. One such initiative is the “*Comer en compañía*” (Eating in Company) project, run by *Mujeres Supervivientes* (Survivor Women). Every Wednesday, the house opens its doors as a social dining space to feed anyone in need. The coordinators -referred to here by the pseudonyms Valeria and Gema^1^- welcomed the researcher warmly and gave her a tour of the premises. The house consists of several rooms connected to a central courtyard, each dedicated to different activities. It includes a library, a kitchen with a dining area, a conference room, and an office to assist those seeking help.

##### 2.2.3.2 Workshop organization

After familiarizing herself with the location and the coordinators, the researcher collaboratively designed the workshops in a horizontal (non-hierarchical) manner. To promote the workshops, posters were created listing the topics to be covered, schedule, location, collaborating organizations, funding sources, and an email address for registration (open to cis, trans, and non-binary individuals). The first workshop, held on September 28 at 12:00 p.m., addressed gender-based violence. Promotion was done through the social media accounts (Twitter, Instagram, Facebook) of *Mujeres Supervivientes* and the researchers’ personal accounts, where the workshop poster was shared. Interested participants emailed proyectosevilla2021@gmail.com, receiving a confirmation of participation and a request to arrive slightly early to take part in qualitative and quantitative research. For the second workshop (on sexual health, October 26 at 12:00 p.m.), participants were emailed an updated poster. The final workshop took place on March 22 at 12:00 p.m., with its poster also sent via email.

##### 2.2.3.3 Development of qualitative and quantitative analysis

When participants attended the first workshop, they were given a qualitative interview before the workshop began, as well as a survey. At the start of the final workshop, all attendees completed the survey again, and interviews were conducted at the end with those who had participated in the initial interview and attended the last session. For those who missed the final workshop but had participated in the initial interview, follow-up interviews were conducted on a later date at *Casa Pumarejo*. The same procedure was applied to the surveys for participants who had completed the initial survey but did not attend the final workshop.

##### 2.2.3.4 Life project mapping

One of the coordinators, Gema, described the gender-based violence prevention plan for affected women, based on their own methodology. The entire conversation was recorded, and the discussion—held separately from other association members—took place on one of the benches in *Casa Pumarejo’s* courtyard. The conversation lasted about 15 min, during which she explained the association’s work with each woman suffering from gender-based violence. Additionally, she emailed one of the researchers documentation and concept maps outlining their entire support process for affected individuals.

### 2.3 Ethical considerations

This work is part of the project: “Prevention and Protection Against Structural Gender Violence Through the Pumarejo Social Dining Initiative, Seville,” funded under the 2020/2021 Call for Development Cooperation Activities and Projects by the University of Seville. The research protocol was approved by the Coordinating Committee for Biomedical Research Ethics of Andalusia (CCEIBA).

## 3 Results and discussion

### 3.1 Characterization of the study population

Table 1 summarizes the characteristics of the intervention participants. The majority of the sample (65%) were between 20 and 30 years old. All participants were women, except for 10% who identified as non-binary. Most declared themselves as single and without a partner (55%), 75% had university-level education, 40% were professionally inactive at the time, and 85% had no children. In other studies, the average age tends to be around 20 (Duran and Eraslan, 2019; Ibabe et al., 2016), similar to the participants in this intervention—though some research includes older participants, average 40 years (Fiskin and Sari, 2021).

**Table 1:** Participant characteristics.

Sociodemographic data	Number	Percentage (%)
Age		
20–30	13	65
31–40	3	15
41–55	4	20
Sex		
Women	18	90
Non-binary	2	10
Current status		
Currently with a partner	7	35
Not currently with a partner, but has been for <1 year	3	15
Had a partner for >1 year	5	25
Never had a partner	3	15
Other	2	10
Primary education		
University	15	75
Secondary education	4	20
Basic education	1	5
No education	0	0
Current professional status		
Not active	8	40
Active	6	30
Unemployed	4	20
Other	2	10
Marital status with partner		
Single, without a partner	11	55
Single, with a partner	7	35
Married	1	5
Other	1	5
Children		
No	17	85
Yes	3	15

According to qualitative interviews, 100% of respondents had experienced gender-based violence, either directly or through close family members. However, none had received formal training on gender equality or violence; their knowledge came from personal research. These findings are summarized in Table 2. In contrast, Giger et al. (2017) found that 50.24% were unaware of gender violence cases, while 11.20% had been victims. Another study (Vives-Cases et al., 2008) reported 15.2% of the sample experienced gender violence in the past year, and Duran and Eraslan (2019) noted 26.6%. These discrepancies may stem from the inclusion of male participants in those studies.

**Table 2:** Characterization of interviewed participants (Qualitative research).

Characteristics	Number	Percentage (%)
Age		
20–30	1	33
31–40	1	33
41–55	1	33
Sex		
Female	3	100
Non-binary	0	0
Has experienced gender-based violence		
Yes, herself	1	33
Yes, family members or acquaintances	2	66
No	0	0
Training in gender-based violence		
Yes	0	0
No	3	100

In our study, we did not report significant differences in perceptions of gender-based violence based on age. All participants, regardless of age, experienced violence. However, women with some education and community support in the field recognized gender-based violence sooner and better, highlighting the importance of community education programs for the prevention of gender-based violence for women of all ages.

### 3.2 Life project mapping

**Figure 3:**
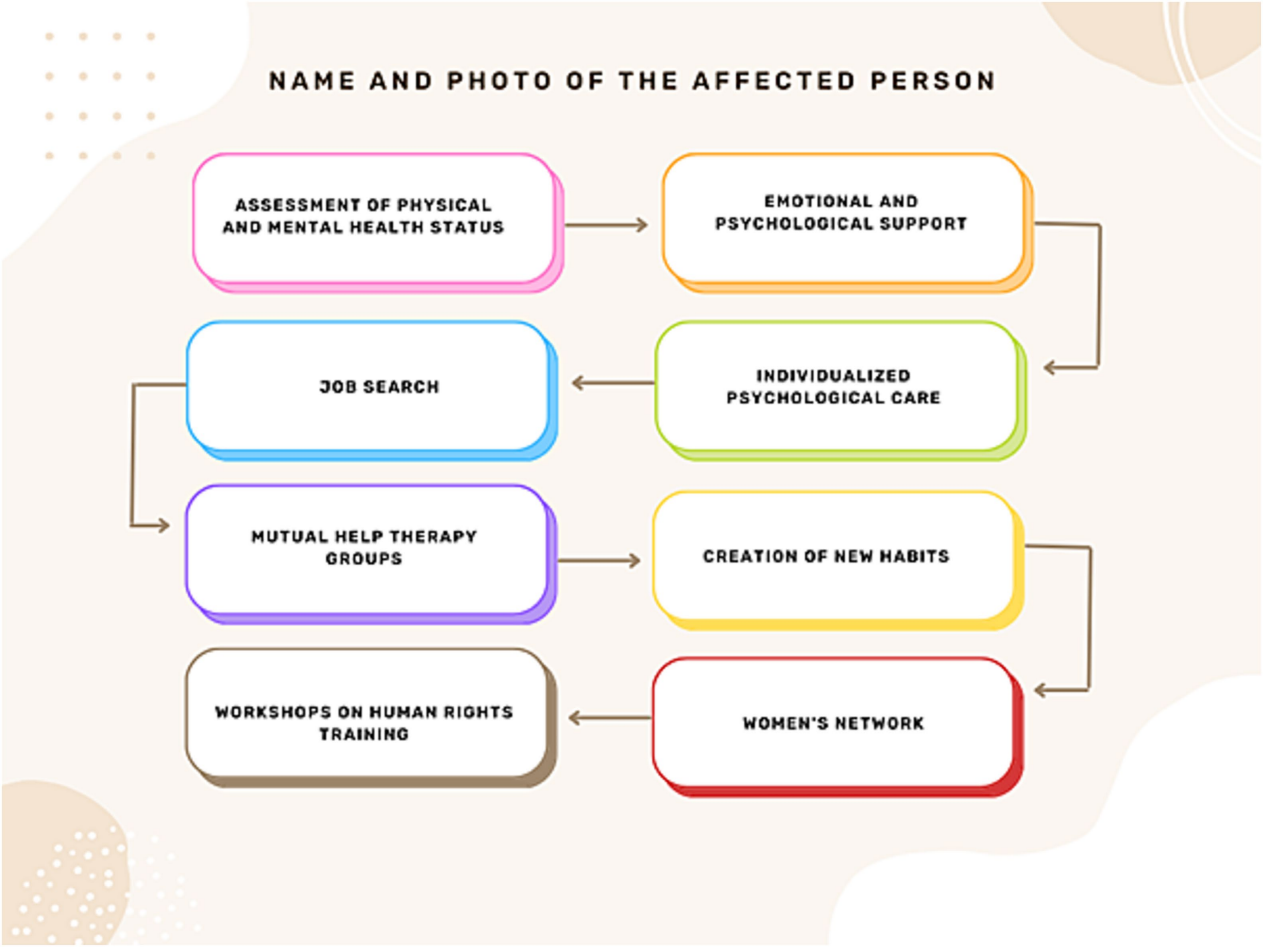
Composition of the life project mapping, provided by *Antonia Ávalos Torres*.

Life Project Mapping is a methodology developed by *Antonia Ávalos Torres*, head of *Mujeres Supervivientes* and a Mexican feminist activist awarded the Silver Rose Prize at the European Parliament in Brussels for her lifelong advocacy for women’s rights. This model is a support and intervention framework rooted in empathy, respect, and the dignity of gender-violence survivors, particularly targeting women paralyzed by fear, distress, or survival mode—those in constant alertness, deprived of sleep and basic self-care. Its foundation is a loving pact with survivors: a therapeutic agreement where women place trust in others. Once this commitment is secured, work begins on rebuilding their lives and breaking cycles of violence. The methodology follows a step-by-step process (Figure 3), outlined below.

**Figure 4:**
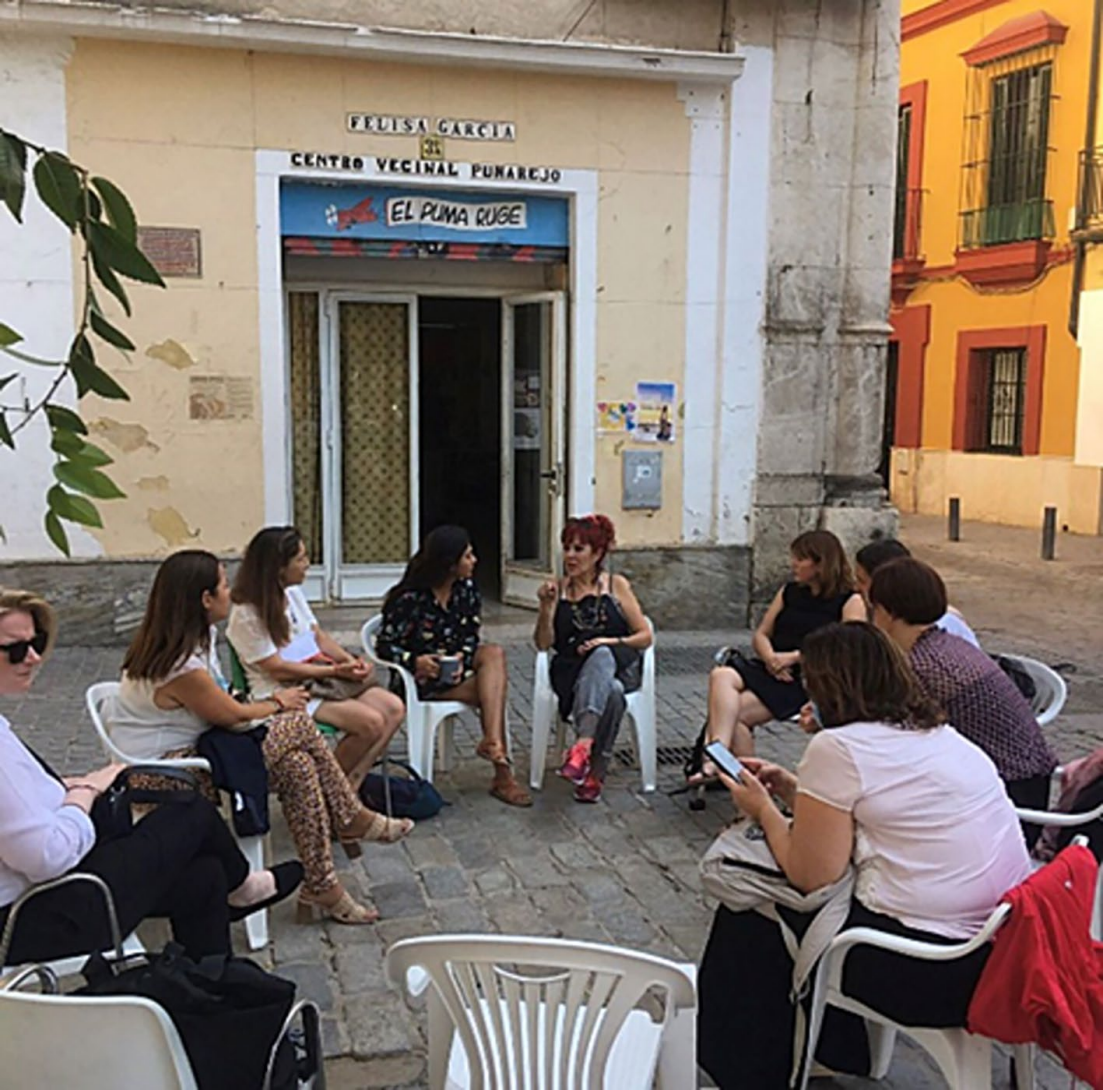
Mutual aid therapy among the leaders of the *Mujeres Supervivientes* association, volunteers, and women suffering from gender-based violence.

First, there must be recognition of this suffering, this lack of self-love, self-esteem, decision making capacity, and self… This process of personal reconstruction is framed within the mapping of the life project. Initially, women experiencing violence must be provided with emotional and psychological support, a risk assessment must be conducted, and their physical and psychological health must be assessed. To this end, they are offered a ride to the hospital if they agree. If they refuse, they are offered emotional support and comforted. The next step is to refer them to psychological care, so that the psychologist can make a professional contract with them so that they commit to attending, and then a schedule of when they should go is established. In addition, some affected women also need to find employment, so they receive help with their resumes, understanding their professional and academic interests to assist them in their job search, and are even escorted to the places where they need to drop off their resumes. Later, they also work in mutual-help therapy groups (Figure 4), so they can begin to learn how to set boundaries with any authority figure, as this requires training.

**Figure 5:**
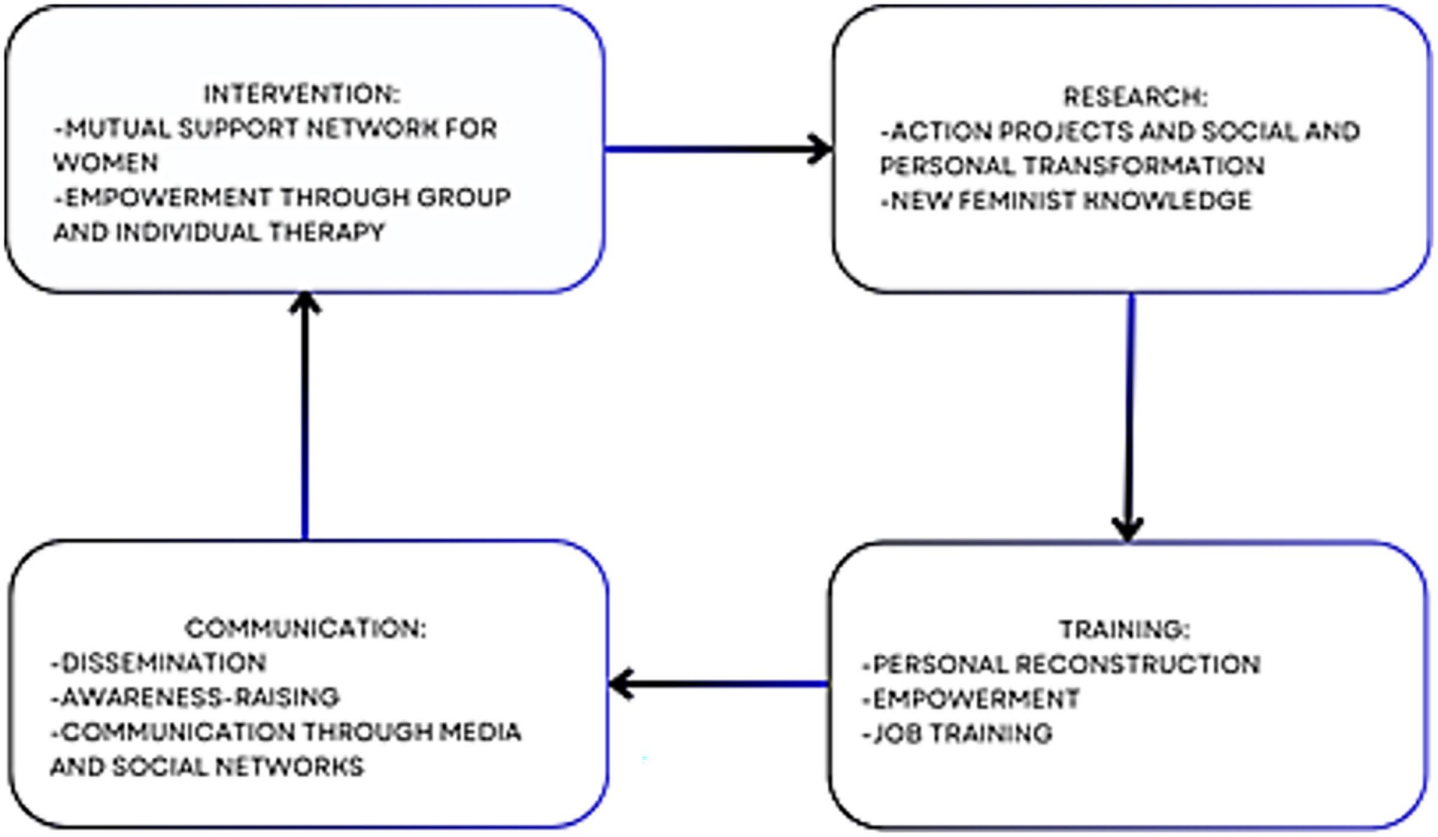
Axes of action, provided by *Antonia Ávalos Torres*.

Depending on their interests, they are taught to create new habits in their lives and to maintain a personal schedule - as long as they do not live with their abuser to avoid putting themselves at risk. This helps them evaluate the decisions they are making, those they are planning, and those they have achieved, in order to rethink their habits, escape that chaos, and plan their life and personal reconstruction. Additionally, a series of workshops on Human Rights are conducted to explain that patriarchal violence is a structural issue stemming from a sexist and patriarchal society. These workshops address abusive power dynamics between men and women and the socialization of romantic love. By understanding this, women begin to comprehend gender roles and recognize that the abusive relationships they have experienced are part of a larger structure that creates a subjectivity of vulnerability and the need for the other to “validate, care for, and protect them at the cost of their own life, dignity, and safety, reducing them to nothing.” Thus, there is also an understanding of the social and political dimensions. In fact, women who have experienced violence often carry tremendous guilt for having “allowed” it, without understanding why - whether due to social conditioning, family influences, media messaging, or the lifelong socialization that portrays women as fragile, sweet beings without autonomy, denied the freedom to make decisions about their bodies, sexuality, relationships, careers, leisure, or family life. These workshops thus serve to alleviate guilt and create a powerful network connecting the political (generating spaces for debate and reflection) with the personal (psychological and emotional). Similarly, self-defense workshops, art workshops, dance workshops, etc. are offered because this mapping is complemented by another action plan called “How to Cultivate Self-Love?” which focuses on nurturing and aesthetic appreciation of one’s being, body, and existence - learning to care for the body, accept it, and love oneself. Then, on the emotional and subjective level, there are other ways to cultivate the mind, such as through art, spending time with friends, literature, cinema, music, therapy - all to help them understand their personality and recognize each of its parts, as this helps restore self-love. All of this is complemented by women’s networks, which are crucial for carrying out the life project. The methodological framework (Figure 5) is based on four key areas: (i) intervention: supported by mutual aid networks and various survival alliances among women, as well as personal reconstruction and empowerment through group work and individual therapy; (ii) research: action-oriented research projects for social and personal transformation, grounded in feminist epistemology to generate new knowledge for social change; (iii) training: a process of personal reconstruction, empowerment, and job training for women; and (iv) communication: projects focused on awareness-raising, advocacy, and communication through various media and platforms like social networks.

Another proposal of the project is learning to love among women, developing a “sisterly” love. As Antonia explains:

Because to the extent that one loves her sisters, does things with them like dancing, parties, trips, moments of laughter, movies… she isn’t hungry for toxic romantic love because a partner isn’t everything, since by having the love of her sisters and loving them in return, that part of love is satisfied.

It’s worth noting that the creator of this methodology states that the model (Figure 5) works and it is a very original and effective way to intervene. Moreover, it’s implemented by professionals trained at Universities and Social Services in Seville.

### 3.3 Evaluation of prior knowledge and lived experiences of violence

According to surveys, 75% of respondents had received training on gender equality and gender violence prior to the workshops because they were students of vocational training or degree programs related to social issues like gender violence, feminism, homophobia… compared to 25% who had not. Notably, all reported having experienced gender violence, whether directly (30%), through a family member (60%), or through an acquaintance (70%), meaning all had encountered gender violence from some perspective. Participants with prior knowledge were typically students of gender equality-related programs. While these percentages offer valuable insights into the patterns observed within this particular group, it is imperative to acknowledge that they are not generalizable beyond this sample. This method allows for a clear exposition of the observed phenomena without implying statistical significance or representativeness that the sample size cannot support.

In the qualitative study, none had received formal training on the subject, and any knowledge came from personal research. Mónica commented:

No, but since I’ve been a victim of gender violence, I’ve taken it upon myself to read on my own[…] what I’ve done is read extensively online and talk with women who’ve had similar experiences

In qualitative interviews about their experiences with gender violence, all had encountered it directly or indirectly. When asked how they responded, none took action - some because they did not know what to do as children, as Lucía explained:

Well, I didn’t know what to do because I was very young, so I tried to help, but obviously when it’s a physically large man and you’re like 4-5-6 years old, there’s really nothing you can do.

In Beatriz’s case, because the victim asked her not to intervene:

No, because my sister told me not to get involved.

Today they would know what to do and whom to turn to - trusted individuals rather than official institutions or police authorities, which they distrust, as Mónica explained:

I tried approaching prevention organizations, but I felt so much shame that I couldn’t continue, I couldn’t even talk about it because in some cases I encountered people, mainly men, who didn’t understand I was experiencing gender violence. […] What helped me greatly was talking with other women about what happened […] because I discovered this made women in my group, my circle, share their experiences and together we could recognize it.

The thematic structure developed through the rigorous coding process illuminated several key areas of insight. For instance, all women experienced violence at some point of their lives. This theme, consistently identified during the coding process, provides a rich understanding of the normalizing of violence by women in society. The analytical framework employed allowed for a nuanced interpretation of these themes, moving beyond mere description to offer deeper insights into the structural violence suffered by women nowadays, mostly in vulnerable conditions.

#### 3.3.1 Evaluation of romantic love myth recognition

Using the AMIVAW scale, most participants agreed in their responses. In this study, participants were quite good at detecting toxicity in romantic love, with an average score of 23.05 (range 15–105; closer to 15 indicates less internalization of romantic love myths). Other studies using this scale show participants do not detect romantic love myths as effectively (Courtois et al., 2021) and do not recognize violence in survey statements. This may be due to different study populations including men and women (Giger et al., 2017).

Qualitative analysis examined transmission models of these myths and prejudices, with all agreeing gender violence occurs across all social classes and ages, as Mónica noted:

No, in fact, gender violence is part of the patriarchal system and doesn’t distinguish between social classes, religion, or backgrounds - whether you live in the mountains, a village, or come from a wealthy family, it doesn’t matter.

Or Beatriz:

I think at all ages. It may seem more prevalent among young people, but it continues at all ages.

Regarding signs of a gender-violent man, all described him as “dominant, aggressive, controlling…” Lucía described it thus:

When he controls, for example, your clothing, setting curfews for when you can leave or return home, controlling your social media…

Herein, it is showed that in this kind of love, we lack from the decision/commitment component of the triangular theory of love. This component can be essential in love relationships, above all for getting through hard times and for returning to better ones, when the communication is indispensable (Sternberg, 1986).

#### 3.3.2 Recognition of sexism and violence

**Figure 6:**
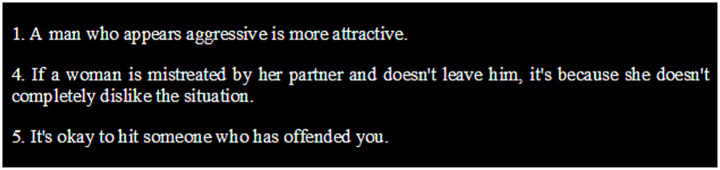
Statements from the questionnaire on justification of sexism and violence.

95% of participants could perfectly identify sexist and violent attitudes. Only 5% did not fully disagree with three items (Figure 6). Regarding the qualitative analysis, knowledge, beliefs, and stereotypes about gender-based violence were assessed. For all participants, gender-based violence is that which targets women, as stated by Lucía or Mónica:

I understand it as any type of violence carried out because someone is a woman.

I understand gender-based violence as any act that infringes upon the personal freedom of women, in this case, anything that goes against the body, ideas, or freedom.

All of them believe there are various types of violence:

Emotional or economic, psychological or… I don’t know what else, but I know there are many.

There is economic, psychological, sexual, and physical violence.

Regarding the origin, they associate it with the patriarchal and structural society:

Well, I believe the origin of gender-based violence comes from a patriarchal system, where there are roles assigned to men and women, which have been upheld for many generations; in which women have a secondary role in society.

This is also linked to Bourdieu’s notion of symbolic violence, as these women “normalize” this structure (Bourdieu and Passeron, 1990), where male dominant group subordinates women (Bourdieu, 1977), leaving them with this violence that produces frustration, alienation and dependency, between others, according to Bourdieu (Lamont and Lareau, 1988). Also, it is demonstrated that structural heteropatriarchy is associated with increased risk of reporting maternal morbidities (Everett et al., 2024).

However, none of them know how many victims of gender-based violence there have been so far this year in Spain, although Mónica states “too many”:

Well, I don’t know, but I would imagine, at least 5 out of 10 women throughout their lives have suffered some type of violence.

And the signs they know how to identify are similar; some include “*man’s control over the woman*” “*isolation from her friends and family members”* or *“physical signs like a bruise*.”

#### 3.3.3 Difference between hostile and benevolent sexism

The participants demonstrated excellent ability to detect hostile sexism, and most could also identify benevolent sexism, though a small percentage showed some difficulty in perfect recognition. This finding aligns with other studies (Rodríguez-Burbano et al., 2021). However, when male populations participate (Ibabe et al., 2016), they also exhibit challenges in detecting hostile sexism.

Notably, in the qualitative analysis, participants were asked about their emotional responses to gender-based violence: all reported feeling “*sadness*” and Mónica additionally mentioned experiencing “*rage and disgust*.” These emotional impacts often lead women to migrate to other countries, as documented in Willers (2016) research featuring testimonies from Central American migrant women in transit through Mexico. These women describe the violence they endure simply for being female and their resulting emotions: “*sad*,” “*furious*,” “*afraid*.”

#### 3.3.4 Knowledge gained after the intervention

**Figure 7:**
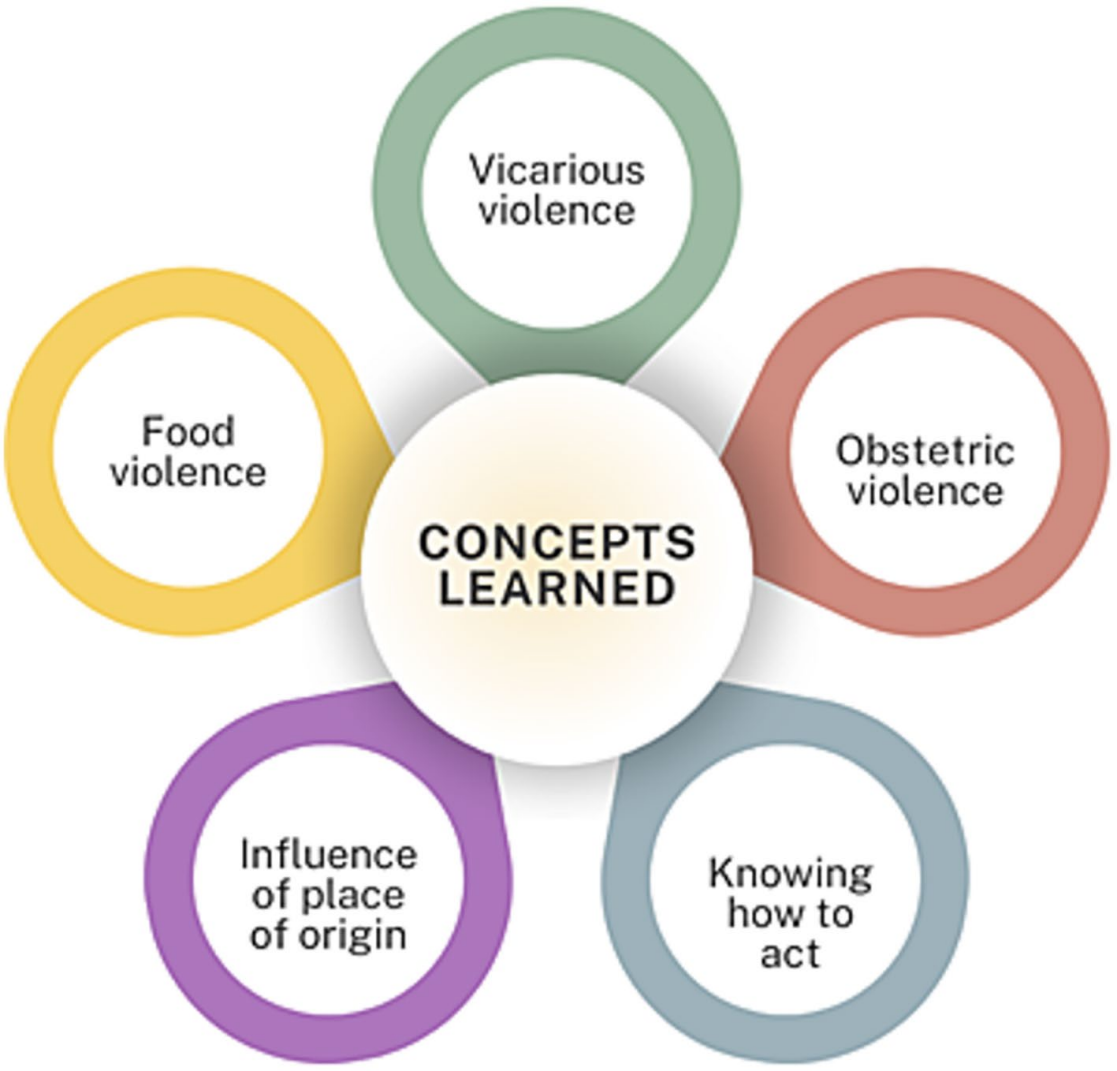
Diagram of new knowledge acquired by participants.

50% of final surveys showed no significant differences compared to initial surveys. 25% differed on one item from the romantic love myths scale (AMIVAW): “*After a complaint of abuse, men are left unprotected by the law*.” The remaining 25% showed several significant differences across various items on the ISA scale. It’s particularly noteworthy that some participants had no prior training in gender violence or equality before the workshops, suggesting these sessions helped them identify sexist behaviors and attitudes. Through qualitative interviews, we identified new concepts learned by participants (Figure 7). Lucía learned to recognize more types of violence, including obstetric violence -violent practices by healthcare professionals during pregnancy, childbirth, and postpartum- (Lima et al., 2021), which she had not known existed. Beatriz initially believed young people committed more gender violence, but now understands age is not a factor - violence can occur at any age. She also now knows proper response protocols: “*I would go to the police station to file a report and to associations like Mujeres Supervivientes for help*.” Mónica expanded her understanding to include concepts like vicarious violence -indirect violence through third parties- (Porter and López-Ángulo, 2022) and the link between sexism and eating disorders (Garaigordobil and Maganto, 2013). She also revised her view about social class influences, now recognizing factors like race and origin that increase vulnerability, as shown in studies of migrant versus native women (De and Porte, 2019; Vives-Cases et al., 2008).

All participants positively evaluated the intervention, finding it enriching for sharing diverse lived experiences. The prevention program involved 20 native and migrant participants attending workshops at *Casa Pumarejo*, including volunteers, interns from *Mujeres Supervivientes* Association, and interested community members. The association has successfully implemented a methodology for supporting gender violence survivors. Our study assessed participants’ internalized romantic love myths and perceptions of sexism/violence, then tracked newly acquired knowledge and the training’s practical utility.

#### 3.3.5 Limitations of the study

While the intentionally small sample size of 20 participants was deliberate to facilitate in-depth qualitative exploration and gather rich, nuanced data, it inherently restricts the generalizability of the findings. The insights derived from this cohort represent the perspectives and experiences of these specific women and may not be representative of the broader population from which they were drawn or similar populations. Due to this limited sample, the study was unable to employ inferential statistical analyses, such as hypothesis testing or the calculation of confidence intervals. Consequently, all findings are presented as descriptive percentages, which accurately reflect the observed frequencies within this specific sample but do not permit extrapolation or claims of statistical significance to a wider population. Future research with larger, more diverse samples would be necessary to explore the generalizability of these findings and to employ robust statistical methodologies.

While the rigorous coding process and thematic structure ensured a systematic and transparent approach to data analysis, it is important to acknowledge the inherent subjective element in qualitative research. The interpretation of data, even with careful methodological steps, is ultimately shaped by the researcher’s lens. Efforts were made to mitigate this through researcher reflexivity and regular peer debriefing sessions. Furthermore, the analytical framework, while chosen to allow for emergent themes, necessarily frames the way findings are presented. Future research could explore alternative analytical lenses to potentially uncover different facets of the data.

A significant limitation of this study stems from the absence of a control group. Given the qualitative and exploratory nature of this study, which aimed to gain in-depth understanding of gender-based violence lived experiences of women who live in vulnerable conditions, the use of a control group was not deemed appropriate or feasible. However, the rich, contextualized data gathered through our chosen methodology provided an emic perspective that would not have been enhanced, and indeed could have been compromised, by a forced comparative structure.

Furthermore, this study is subject to selection bias due to its small sample size (n = 20) and the utilization of a snowball sampling technique. This method, while effective for reaching specific social networks, as the one of this study needs, inevitably does not account for women outside this network. Consequently, the findings of this study, while offering valuable insights into the experiences of the interviewed women, cannot be generalized to the broader population. Readers should interpret the results as context-specific understandings rather than universal claims.

## 4 Conclusion

This study evaluated a community intervention strategy for preventing gender violence among people who live in vulnerable conditions. The program involved 20 migrant and native women connected to *Casa Pumarejo*’s social dining program through volunteering, internships, or workshop participation. The study revealed that association volunteers employ a unique, effective methodology called Life Project Mapping for supporting gender violence survivors.

All workshop participants had experienced gender violence during their lifetimes. While 75% of survey respondents had prior gender violence training (through gender studies), none of the qualitative interviewees had such background. These findings largely align with existing literature on gender-based violence, particularly concerning the pervasive influence of romantic love myths, ambivalent sexism, and structural vulnerability. The workshops successfully educated all interested participants. Post-intervention, all acquired new knowledge and valued the workshops for improving violence detection/prevention skills. The “*Life Project Mapping*” intervention contributed to significant changes in participants’ knowledge, attitudes, and behaviors through: (i) the recognition of suffering and personal reconstruction, (ii) emotional and psychological support, risk assessment, and referrals to professional care, (iii) mutual-help therapy groups, (iv) workshops on Human Rights and the deconstruction of patriarchal violence and romantic love socialization and (v) cultivating self-love through art, literature, therapy, and women’s networks. The concept of “*sisterly*” love as a counter-narrative to toxic romantic love represents a powerful social and emotional mechanism for building resilience and reducing vulnerability.

Similar strategies could prevent gender violence cases or enable early detection before escalation, particularly reaching people who live in vulnerable conditions like migrant women. Despite the valuable insights generated, this study has several limitations concerning its methodology, sample, and context, which warrant careful consideration in interpreting and generalizing the results. Future initiatives should consider developing parallel programs for male populations to increase global awareness of gender violence issues.

## Data Availability

The original contributions presented in the study are included in the article/supplementary material, further inquiries can be directed to the corresponding author/s.

## Footnotes

^1^In this research, it was decided to use fictitious names for the participants and interviewees in order to preserve their privacy and anonymity.
